# Nutritional and Antioxidative Benefits of Dietary Macroalgae Supplementation in Weaned Piglets

**DOI:** 10.3390/ani14040549

**Published:** 2024-02-07

**Authors:** Anna Czech, Katarzyna Woś, Siemowit Muszyński, Ewa Tomaszewska

**Affiliations:** 1 Department of Biochemistry and Toxicology, Faculty of Animal Sciences and Bioeconomy, University of Life Sciences in Lublin, 20-950 Lublin, Poland; anna.czech@up.lublin.pl; 2 Department of Biophysics, Faculty of Environmental Biology, University of Life Sciences in Lublin, 20-950 Lublin, Poland; siemowit.muszynski@up.lublin.pl; 3 Department of Animal Physiology, Faculty of Veterinary Medicine, University of Life Sciences in Lublin, 20-950 Lublin, Poland

**Keywords:** antioxidant activity, dietary supplement, macroalgae, piglets

## Abstract

**Simple Summary:**

This study was conducted to investigate the health benefits of incorporating brown macroalgae into the diets of young piglets. Different amounts of macroalgae were added to piglet feed, and the effects on their digestion and health were observed. It was found that the inclusion of macroalgae enhanced the piglets’ ability to digest fat and had a positive impact on their blood cholesterol levels. Additionally, an increase in the body’s defense against oxidative stress, a condition that can cause cellular damage, was noted. This study suggests that macroalgae, a natural seaweed supplement, could be beneficial in piglet diets, aiding in digestion and health improvement. This research is significant as it explores natural methods to enhance animal health and nutrition.

**Abstract:**

This study explores the effects of dietary brown macroalgae (*Ascophyllum nodosum*) inclusion on digestibility and blood biochemical indices and redox markers in piglets fed diets with varying levels (0%, 0.6% and 1%) of macroalgae from 18 to 64 days of age. Macroalgae significantly influenced lipid profiles, reducing total cholesterol levels (quadratic contrast *p* = 0.001) and demonstrating an increase in high-density lipoprotein cholesterol levels, particularly with 1% macroalgae inclusion (linear contrast *p* < 0.001), with a decrease in low-density lipoprotein cholesterol in both macroalgae-supplemented groups (linear contrast *p* = 0.001). Additionally, macroalgae had a positive impact on the activities of antioxidative enzymes (ferric-reducing ability of plasma, superoxide dismutase, reduced glutathione) and reduced lipid peroxidation products (lipid hydroperoxide, malondialdehyde) in the blood, liver tissue, and intestinal epithelium of the ileum, suggesting enhanced antioxidative defense mechanisms. These changes were dose-dependent; in blood plasma, they exhibited both a linear and quadratic response, while in the tissues, the response was primarily linear. Additionally, an increase in the digestibility of crude fat in macroalgae-supplemented groups was observed (linear contrast *p* < 0.001), highlighting their potential role in improving nutrient absorption and digestion. The study findings emphasize the health benefits of natural, seaweed-based additives in diets, particularly in managing oxidative stress and improving lipid profiles, and highlight the potential of macroalgae as a natural dietary supplement to improve antioxidant systems and lipid metabolism in piglets.

## 1. Introduction

Macroalgae, commonly referred to as seaweed, are plant-like organisms divided into three primary groups based on pigmentation: brown seaweed (*Phaeophyceae*), red seaweed (*Rhodophyceae*), and green seaweed (*Chlorophyceae*) [1]. Seaweed, with its extensive taxonomic diversity, is a significant source of bioactive substances. This has spurred numerous studies to explore the potential health benefits of seaweed. The incorporation of macroalgae in diets has shown promising effects in managing conditions like cardiovascular diseases, rheumatoid arthritis, diabetes, and obesity due to their anti-inflammatory properties. Notably, the chemical compounds in macroalgae with anti-inflammatory potential do not exhibit the side effects often associated with non-steroidal anti-inflammatory drugs [2]. Macroalgae are also valued as food additives due to their rich content of carbohydrates, polyphenols, peptides, carotenoids, vitamins, minerals, and a balanced presence of n-3 and n-6 polyunsaturated fatty acids (PUFA) [1]. The brown hue of *Ascophyllum nodosum* is primarily due to its high carotenoid fucoxanthin content, which overshadows other pigments like β-carotene, violaxanthin, diatoxanthin, and chlorophyll. It is also characterized by polysaccharides such as laminarin, fucoidans, and alginates, along with a cellulose-rich and acid alginic cell wall [3], and shows anticancer, anticoagulant, antibacterial, antiviral, antifungal, immunostimulating, antioxidant, and anti-inflammatory properties [4,5].

Previous studies on microalgae supplementation concerned weaning piglets at different ages: 4, 5, or 3 weeks of age, or pigs in the grower–finisher period and rather concerned aspects related to intestinal microflora, immune function, or edible tissues [6,7,8,9,10]. In this study, weaning piglets were chosen. This phase is critical as piglets are particularly vulnerable to reduced immunity due to oxidative stress, a key factor in gastrointestinal mucosa disorders. Oxidative stress results from an imbalance between reactive oxygen species (ROS) production and the antioxidant system’s efficiency. Research suggests that the dietary inclusion of macroalgae enhances the effectiveness of the antioxidant system, including reduced glutathione (GSH), superoxide dismutase (SOD) and catalase (CAT), thus reducing ROS levels and strengthening the intestinal barrier against mucosa-related diseases [11,12]. Brown algae, in particular, are known to exhibit the highest antioxidant activity. Moreover, constituents in brown algae, such as terpenoids, polysaccharides, and polyphenols, have been observed to affect adipogenesis, leading to a dose-correlated decrease in cellular lipids [13]. 

Considering the advantageous attributes of algae elucidated earlier, our hypothesis posits that incorporating a macroalgae product into a diet could yield benefits by the improvement of feed utilization, basic biochemical parameters, and antioxidant status in piglets. The novelty of this study stems from its unique combination of macroalgae supplementation, a well-designed experimental setup, a detailed digestibility study, comprehensive sample analysis, and the exploration of oxidative stress markers in both blood and tissues. These elements collectively contribute to the originality and significance of the research in the context of piglet nutrition and health. The study described here is particularly distinctive because it covers the time before and after weaning until the piglets reach an age of 64 days and a body weight of approximately 15 kg. 

Based on the beneficial attributes of algae outlined above, our hypothesis is that the incorporation of *Ascophyllum nodosum*, a macroalgae product, into piglet diets could result in improved feed utilization, basic biochemical parameters, and antioxidant status. This study aims to analyze the feces from piglets fed algae to determine digestibility indices, which are crucial for assessing nutritional efficiency and optimizing feed utilization. Additionally, the study will examine the impact of varying levels of macroalgae in piglet feed on biochemical indices, including blood lipid profiles, and oxidative stress levels measured in blood and tissues.

## 2. Materials and Methods

### 2.1. Animals and Experimental Design

The study was conducted with a cohort of piglets (Landrace and Yorkshire hybrid [LY] × Duroc and Pietrain hybrid [DP] crossbreed), offspring of 15 sows in their second or third lactation cycles. Initially, after parturition, to ensure consistent experimental conditions, piglets were evenly distributed among the sows. This standardization resulted in 14 piglets per litter with an equal proportion of female (gilts) and male (barrows) piglets in each litter. After the standardization of the litters, all 210 piglets were ear-tagged for clear identification and assignment to their respective replicate. Subsequently, the sows with their litters were then randomly divided into three groups, each comprising piglets from five sows per experimental group. The experiment began when the piglets were 18 days old (10 days before the planned weaning) and were introduced, at the litter level, to their respective experimental diets.

The control group (the C group) of piglets received a standard pre-starter mix without any macroalgae addition. Piglets in the A-0.6 and A-1 groups received a mix similar to the control group, but with wheat replaced by ground dried brown macroalgae (*Ascophyllum nodosum*; European Protein, Bække, Denmark) in amounts of 0.6% (the A-0.6 group) or 1% (the A-1 group) of feed dry matter. Macroalgae, with a dry matter (DM) content of 880 g/kg, contained a declared 7 g/kg DM crude protein, 30 g/kg DM crude fat, 40 g/kg DM crude fibers, 210 g/kg DM crude ash, 30 g/kg DM sugars and 450 g/kg DM soluble fibers. 

Piglets were housed with their sows from the beginning of the experiment until the 28th day of life, at which point they were weaned. Then, a total of 150 piglets, with 50 piglets in each group (10 piglets from each litter, matched for body weight and sex, with 25 gilts and 25 barrows in each group) were transferred to one pen per treatment in the weaner unit and continued on the same dietary treatment based on the same pre-starter diet. The experiment lasted until the piglets were 64 days old and reached an approximate weight of 15 kg.

Throughout both stages of the experiment, feed mixtures and water were provided to the piglets ad libitum. The nutritional composition of the applied pre-starter diets adhered to the guidelines set by the NRC [14] and were designed to be iso-energetic and iso-protein across treatments (Table 1), with a basal barley–wheat based diet containing 18.75% crude protein, 6.92% crude fat, 3.49% crude fiber, and providing 14.0 MJ/kg of metabolizable energy (ME).

### 2.2. Collection of Feces and Analysis of Digestibility Indices

The digestibility study analysis was conducted when the piglets were 50 days old. To calculate the nutrient digestibility coefficient, the acid insoluble ash (AIA) method was used, with silicon dioxide (SiO_2_) added as a marker to the feed mixtures (2 g/kg) before granulation [16]. Six piglets (three gilts and three barrows) from each treatment group were randomly selected. The selection included one piglet from each of the five replicate litters, identified by ear tags, and an additional piglet selected randomly from the remaining piglets to maintain an equal number of gilts and barrows. The selected animals underwent a 6-day adaptation isolation during which they were fed the marked feed [17]. Subsequently, fecal samples were collected and weighed from each pig over the next four consecutive days, at the same time each day. The collected feces, approximately 20% from each pig, were placed in plastic bags and stored at a temperature of +4 °C until transported to the laboratory. The fecal samples collected over 4 days were pooled by pig. In the dried and homogenized samples of feed and collected feces, the content of basic nutrients: crude protein, crude fat, and crude fiber were analyzed according to AOAC [18] methods (procedures 990.03, 920.39, 978.10, respectively). To estimate water-soluble polysaccharides (sugar, starch), the content of nitrogen-free extract was also calculated.

The apparent total tract digestibility coefficients (ATTD, %) of the nutrients were calculated using the following formula [19]:

ATTD = (1 − (N_f_ × M_d_)/(N_d_ × M_f_)) × 100%, (1)

where N_f_ represents the concentration of the nutrient in the feces, M_d_ represents the dietary concentration of the marker, N_d_ represents the dietary concentration of the nutrient, and M_f_ represents the concentration of the marker in the feces (all values expressed in g/kg DM).

### 2.3. Sample Collection

On postnatal day 64, selected piglets undergoing the digestibility study were euthanized through electrical stunning followed by exsanguination. Whole blood was drawn into heparinized tubes to prevent clotting. The samples were then centrifuged at 1400× *g* for 10 min at 4 °C to obtain plasma, which was aliquoted into polypropylene tubes and stored at −20 °C for analysis the subsequent day. Immediately post-exsanguination, the abdominal cavity was opened, and segments of the ileum, each approximately 2 cm in length, were excised and thoroughly rinsed with saline to clear any intestinal content. Concurrently, samples from the right lobe of the liver were dissected. All tissue samples were harvested within 5 min of the animal’s death to ensure preservation of biological markers. The collected ileum and liver samples were instantly placed into storage tubes and snap-frozen in liquid nitrogen. They were then stored at −86 °C until further analysis.

### 2.4. Blood Analysis

Blood plasma was subjected to a series of analyses to determine levels of total protein (TP), albumin (ALB), urea (UREA), creatinine (CREAT), uric acid (UA), and bilirubin (BIL). Lipid profiles were assessed by measuring triglycerides (TG), total cholesterol (TCH), high-density lipoprotein cholesterol (HDL), and low-density lipoprotein cholesterol (LDL). The analysis also included calculating the percentage of HDL in relation to total cholesterol (% HDL). The enzymatic profile was evaluated by analyzing alkaline phosphatase (ALP), alanine aminotransferase (ALT), aspartate aminotransferase (AST), lactate dehydrogenase (LDH), and gamma-glutamyltransferase (GTP). These analyses were conducted using colorimetric methods on a Mindray BS-120 automatic biochemistry analyzer (Bio-Medical Electronics, Shenzhen, China), employing commercial ready-to-use test kits from Alfa Diagnostics (Warsaw, Poland) and Cormay Diagnostics (Lublin, Poland). The accuracy of all analyses was confirmed using multiparametric control serum (Alfa Diagnostics, Warsaw, Poland) [20]. 

### 2.5. Determination of Oxidative Stress Markers

The lipid peroxidation products, lipid hydroperoxide (LOOH) and malondialdehyde (MDA), were determined using the methods described by Gay and Gebicki [21] and Esterbauer and Cheeseman [22], respectively. The activities of antioxidative enzymes, including the ferric-reducing ability of plasma (FRAP), catalase (CAT), reduced glutathione (GSH), ascorbic acid, and superoxide dismutase (SOD), were measured spectrophotometrically following the established procedures [23,24,25,26,27]. The same methods used for blood plasma were applied to assess oxidative stress markers, including LOOH, MDA, CAT, GSH, and SOD, in intestine and liver tissues.

### 2.6. Statistical Analysis

Statistical analyses were performed using Statistica software (v. 13.3, TIBCO Software Inc., Palo Alto, CA, USA). Data normality and homogeneity of variances were assessed using the Shapiro–Wilk and Levene tests, respectively. A one-way ANOVA was utilized, designating treatment as the fixed factor and individual pigs as the experimental units. Post hoc comparisons of treatment means were conducted using Tukey’s HSD test. Orthogonal polynomial contrasts were employed to evaluate the linear and quadratic effects of varying macroalgae levels on specific response variables. For all tests, a significance threshold of *p* < 0.05 was established.

## 3. Results

A statistically significant linear increase (*p* < 0.001) in the digestibility coefficient of crude fat was observed in the A-1 group compared to the control, indicating enhanced digestibility with macroalgae supplementation (Table 2). The apparent digestibility coefficient of nitrogen-free extract was significantly higher in the A-0.6 group compared to A-1 (*p* < 0.05), with a notable linear effect of increasing microalgae level (*p* < 0.05). No significant impact of macroalgae on the digestibility coefficients of crude protein and crude fiber was detected.

Table 3 presents the basal biochemical indicators in piglet plasma. Total protein (TP) levels showed both linear and quadratic effects (*p* < 0.001 for both), with the highest levels in the control group, followed by A-1, and the lowest in A-0.6 (*p* < 0.001). Albumin (ALB) levels also demonstrated linear and quadratic effects (*p* < 0.01 and *p* < 0.001, respectively), with the A-0.6 group exhibiting lower ALB levels (*p* < 0.001) and no significant difference between the control and A-1 groups. Urea levels were significantly higher in the C and A-0.6 groups compared to A-1, showing strong linear (*p* < 0.001) and weak quadratic (*p* < 0.05) effects. No impact of macroalgae on creatinine, uric acid, and bilirubin was found. 

Enzyme activities (Table 4) varied significantly with macroalgae inclusion, displaying strong linear (except for ALP) and quadratic effects (*p* < 0.001). Both macroalgae-supplemented groups showed increased activities of ALT and AST and decreased GTP activity. ALP activity in the A-1 group was significantly higher than in the control group (*p* < 0.001). Lactate dehydrogenase (LDH) activity was lowest in A-0.6, followed by the control, and highest in A-1 (*p* < 0.001).

While no effect of macroalgae on triglycerides was observed, 0.6% macroalgae inclusion led to a reduction in total cholesterol (TCH) compared to the C and A-1 groups (*p* = 0.003 and *p* < 0.001 for treatment and quadratic contrast, respectively) (Table 5). High-density lipoprotein cholesterol levels increased in the 1% macroalgae group (*p* < 0.001 for both treatment and linear contrast), while both macroalgae-supplemented groups showed reduced LDL compared to the control (*p* = 0.003 for treatment and quadratic contrast and *p* < 0.001 for the linear effect). Both macroalgae groups also exhibited a reduced %HDL (*p* < 0.01 for the linear and quadratic effects).

Table 6 presents oxidative stress markers in piglets’ blood plasma. Lipid peroxidation markers, LOOH and MDA, were significantly reduced in the 1% macroalgae group, which also showed increased SOD activity (*p* < 0.001 for all effects). Ferric-reducing ability of plasma (FRAP) levels and GSH activity were elevated in both macroalgae groups (*p* < 0.001 for all effects), with no difference between A-0.6 and A-1 for FRAP, while GSH activity was highest in A-1. No impact on CAT and ascorbic acid was noted.

Oxidative stress markers in the liver and intestine (Table 7) showed significant reductions in LOOH and MDA in the A-1 group for both tissues (*p* < 0.01 for the treatment and linear effects for LOOH and *p* < 0.001 for the treatment and linear effects for MDA). In both tissues, GSH activity increased with macroalgae inclusion, being highest in A-1 (*p* < 0.001 for the treatment and linear effects). Liver SOD activity increased with macroalgae level, peaking in the 1% group (*p* < 0.001 for the treatment and linear effects). In the intestine, only the A-0.6 group showed higher SOD activity compared to the control (*p* < 0.01 for the treatment, linear, and quadratic effects). Catalase activity in the intestine increased with macroalgae level, with the highest activity in A-1 (*p* < 0.01 for the treatment and linear effects), while no impact on liver CAT activity was observed.

## 4. Discussion

Numerous studies have shown that certain types of algae can positively impact lipid metabolism, leading to improvements in lipid profiles, especially in individuals with hyperlipidemia [28]. Algae contain various bioactive compounds, including dietary fibers, polysaccharides, and specific types of lipids such as omega-3 fatty acids, which offer many beneficial properties, like lowering LDL and triglycerides, and increasing HDL [29,30] levels.

In the present study, it was found that the inclusion of a lower percentage of macroalgae (0.6%) contributed to lowering the level of total cholesterol, which was not achieved with the supplementation of 1% of algae. The inclusion of both 0.6% and 1% of macroalgae contributed to a significant increase in the HDL cholesterol fraction while reducing the LDL cholesterol fraction. Polyphenols extracted from brown algae have shown their impact on lipid metabolism, assessing the potential benefits of algae polyphenols in managing lipid profiles [31,32]. The favorable changes in the serum lipid profile observed in the groups of piglets receiving the macroalgae supplement suggest the possibility of using them in preventing cardiovascular diseases. This effect may be related to the structure of the polysaccharides present in macroalgae and their effect on the increase in bile acid binding capacity [33].

The observed mechanism is thought to be attributed to soluble fibers classified as polysaccharides, believed to impede the reabsorption of bile salts from the small intestine into the enterohepatic circulation. As a result, excess bile salts are excreted in the feces, leading to a depletion of the bile salt pool, which in turn results in an increased breakdown of cholesterol in hepatocytes. Consequently, the liver must produce more bile salts from cholesterol, which can lead to a reduction in circulating cholesterol levels. This mechanism is one of the ways in which soluble fiber, including that from algae, can help lower cholesterol levels and positively impact cardiovascular health. Additionally, cholesterol esters are metabolized, and the synthesis of LDL membrane receptors takes place, thereby increasing the uptake of LDL from the bloodstream [34]. Furthermore, the hypocholesterolemic effect of macroalgae may be related to altering the lipid profile by modulating high-affinity lipoprotein metabolism receptors [35,36,37].

In addition to its hypocholesterolemic action, macroalgae are also attributed hepatoprotective properties, which are associated with the presence of fucoidan. In the present experiment, the activity of liver enzymes increased in animals supplemented with macroalgae, but it should be noted that the obtained values fell within the range considered as reference values [38]. This might be related to the age of the animals since young organisms are more sensitive to the presence of substances such as polyphenols or phlorotannins found in macroalgae. Therefore, the absence of pathological changes in liver enzyme activity, along with improved antioxidative indicators in the liver (as discussed later in the discussion), demonstrates the positive impact of algae on liver function.

In the present study, a decrease in total protein level was detected, similar to the findings in the study by Poulose et al. [39], which was carried out on rats supplemented with phytosterol extracted from the seaweed *Gelidium spinosum*. This could be related to increased insulin activity, which inhibits protein degradation and enhances the uptake of amino acids, suggesting potential anti-diabetic properties. Insulin plays a key regulatory role in amino acid metabolism, and amino acids, in turn, affect insulin action in a bidirectional way by regulating glucose and protein metabolism [40]. Additionally, in the study by Poulose et al. [39], a reduction in urea and creatinine levels was observed, consistent with the findings of the present study. Urea and creatinine are markers of kidney dysfunction, and the results obtained may indicate the potential of macroalgae to protect against the effects of diabetes.

Macroalgae thrive in challenging environmental conditions, making them susceptible to continuous oxidative stress [41]. To protect themselves from stressful conditions, brown algae produce numerous compounds with strong antioxidant properties, such as fucoidan, polyphenols, and carotenoid pigments, which help reduce the effects of oxidative stress [42]. The present study showed the potential of macroalgae in enhancing the antioxidant system in piglets. At a higher dose of macroalgae, a higher activity of redox enzymes (e.g., SOD, GSH) in the serum was achieved. Similar results were obtained when studying the activity of redox enzymes in the ileum and liver. However, significantly higher CAT activity was only observed in the intestine, not in the liver or serum. These findings align with the studies by Reshma et al. [43], where mice received agar extracted from brown algae *Laminaria digitata*. It was observed that *L. digitata* exhibited stronger antioxidant effects at a concentration of 200 µL of agar compared to 50 µL, as evidenced by the increased activity of enzymes such as SOD, GSH, and CAT at higher agar concentrations [43]. Furthermore, research by Wan et al. [44], conducted on piglets weaned using AOS (alginate oligosaccharide) derived from brown algae, showed a 20% increase in SOD activity and a 37% increase in CAT activity in the serum compared to the control group.

The occurrence of oxidative stress in the presented weaned piglets, which can result from a sudden change in diet, hygienic conditions, and the environment [45], may be indicated by a high level of reactive oxygen species, leading, among other things, to lipid peroxidation. One of the adverse effects of lipid peroxidation is the generation of LOOH, and consequently, MDA. Malondialdehyde is the most mutagenic product of peroxidation and is commonly used as a marker to assess the level of oxidative stress [46]. In the present study, a significant decrease in MDA and LOOH was observed in piglets receiving macroalgae in their diets, regardless of the dosage. The reduction in the levels of final lipid peroxidation products was noted not only in the serum of piglets but also in their liver and ileum. These results support the potential involvement of macroalgae in inhibiting lipid peroxidation, thus demonstrating potential antioxidant activity. Similar conclusions were reached by Wan et al. [44,47]. They demonstrated that supplementation with alginate oligosaccharides (AOS) extracted from brown algae contributes to a 26% reduction in toxic MDA levels in the blood serum and a 36% reduction in the small intestine compared to the control group.

In piglets, the weaning period, characterized by high stress levels, is a critical phase leading to decreased immunity and, consequently, increased susceptibility to disease factors [48]. During this period, there is reduced feed intake and decreased utilization of dietary nutrients, resulting in lowered production performance and higher mortality rates [49]. While the apparent digestibility of nutrients, including fats, in suckling piglets is high, around 96%, during the weaning phase, due to the stress associated with separation from the sow, fat digestibility can drop to as low as 65%. The reason for these changes is likely a reduction in the activity of enzymes responsible for proper fat digestion and absorption in the diet, including gastric and pancreatic lipases [50]. 

Analyzing feces to determine digestibility indices is important because it provides valuable information about the efficiency of nutrient utilization by an organism. Digestibility indices help us understand how effectively not only an animal is able to extract nutrients from its diet and absorb them for growth and metabolic processes but how macroalgae could improve this process [51]. Until now, there have been relatively few studies describing the impact of macroalgae on nutrient digestibility, especially in such a sensitive group of animals as piglets. In the present study, we observed a linear increase in raw fat digestibility associated with the dosage of macroalgae. A similar relationship was found by Wan et al. [52] in their research on weaned piglets receiving a mixture containing 100 mg/kg AOS, which resulted in a 10.8% increase in raw fat digestibility compared to the control group. The reason for these results may be the prebiotic effects of polysaccharides and the antioxidant properties that are present in the structure of macroalgae, impacting the digestive processes. Additionally, the influence of macroalgae on improving the integrity of the intestinal mucosa and increasing villus length contributes to the enhancement of small intestine digestive function and results in better nutrient absorption [3,15].

Algae can influence the digestive process in several ways, primarily due to their nutritional composition and bioactive compounds. Algae impact digestion through soluble fibers such as alginate and agar, which can slow down the digestion and absorption of nutrients, helping to stabilize blood sugar levels [53]. Some algae contain enzymes that aid in breaking down complex carbohydrates, making them easier to digest. For example, certain types of brown algae produce enzymes like laminarinase and fucoidanase, capable of breaking down specific carbohydrates [13]. Additionally, algae can have prebiotic effects by promoting the growth of beneficial gut bacteria. This contributes to a healthier gut microbiome, essential for efficient digestion and overall health [54,55]. Algae are also rich in antioxidants, including polyphenols and carotenoids, which help protect digestive tract cells from oxidative damage, maintain gut health, and reduce inflammation, thereby alleviating digestive discomfort [56]. As previously mentioned, certain compounds in algae, such as fucoidan, can influence lipid metabolism. They may help reduce the absorption of dietary fats, with implications for weight management and cardiovascular health [57]. Furthermore, macroalgae supplementation selectively regulates the gut microbiota, increasing the population of beneficial *Bifidobacterium* and *Lactobacillus* while reducing *E. coli* populations. A well-regulated microbiome strengthens the gut barrier, reducing the likelihood of gastrointestinal diseases [44]. However, it is important to note that the effects of algae on digestion can vary depending on the algae type, its specific components, and the method of preparation and consumption.

## 5. Conclusions

This study has demonstrated that incorporating macroalgae into the diets of weaning piglets notably enhances nutrient digestibility, particularly fat, and positively alters serum lipid profiles by increasing HDL and reducing LDL cholesterol. These changes suggest potential benefits in cardiovascular health management. Additionally, the study highlights the antioxidative benefits of macroalgae, evidenced by a reduction in lipid peroxidation markers and increased activity of redox enzymes in various tissues. The findings also indicate macroalgae’s potential in improving liver health and aiding in digestive processes through its prebiotic effects and modulation of the gut microbiota. Overall, the inclusion of macroalgae in diets emerges as a promising natural strategy to boost overall piglet nutrition and health.

## Figures and Tables

**Table 1:** Ingredients and chemical compositions of the experimental diets [15].

Item/Diet ^1^	C	A-0.6	A-1
Macroalgae	0	0.6	1
Barley	35	35	35
Wheat	27.89	27.35	26.92
Fermented rapeseed meal	10	10	10
Soybean meal	2.7	2.6	2.6
Soy protein	2.5	2.5	2.5
Potato protein	2.5	2.5	2.5
Fishmeal	3	3	3
Whey powder	4.25	4.25	4.25
Whole milk powder	5	5	5
Vegetable oil	2.69	2.78	2.84
L-Lys (98.5%)	0.76	0.75	0.75
DL-Met (99%)	0.21	0.21	0.21
L-Thr (98.5%)	0.29	0.29	0.29
L-Trp (98%)	0.15	0.15	0.15
Monocalcium phosphate	0.98	0.99	0.99
Sodium chloride	0.36	0.31	0.28
Calcium formate	0.7	0.7	0.7
Iron fumarate 31%	0.25	0.25	0.25
Pigor^®^ flavouring aroma	0.2	0.2	0.2
Mineral-vitamin premix ^2^	0.5	0.5	0.5
Sucram	0.07	0.07	0.07
Calculated values			
ME (MJ/kg)	14.0	14.1	14.2
Calcium (g/kg)	7.41	7.54	7.62
Phosphorus (g/kg)	6.60	6.61	6.60
Determined values			
Dry matter (g/kg)	88.03	88.10	88.07
Crude ash (g/kg)	5.85	5.93	5.90
Crude protein (g/kg)	18.75	18.60	18.55
Crude fat (g/kg)	6.92	7.01	7.05
Crude fibre (g/kg)	3.49	3.50	3.49

^1^ Dietary treatments: 0% (C), 0.6% (A-0.6), or 1% (A-1) of macroalgae addition. ^2^ Mineral-vitamin premix in mg/kg of diet for both pre-starter and starter diets: vitamin A—13,000 IU; vitamin D_3_—2000 IU; vitamin E—165 mg; vitamin B_1_—2.5 mg; vitamin B2—7.0 mg; vitamin B_6_—4.0 mg; vitamin B_12_—0.05 mg; vitamin C—100 mg; vitamin K_3_—3 mg; biotin—0.2 mg; niacin—35 mg; folic acid—1.5 mg; pantothenic acid—21.7 mg; iron (sulphate monohydrate)—180 mg; zinc (oxide)—150 mg; manganese (oxide)—55 mg; selenium (sodium selenite)—0.40 mg; iodine (calcium iodate)—0.60 mg.

**Table 2:** Apparent total tract digestibility coefficients (%) of basic nutrients.

Treatment ^1^	Crude Protein	Crude Fat	Crude Fiber	Nitrogen-Free Extract
C	78.7	70.2 ^b^	53.1	88.1 ^a,b^
A-0.6	78.9	72.6 ^ab^	50.0	88.4 ^a^
A-1	79.5	74.6 ^a^	54.0	87.2 ^b^
SEM ^2^	0.42	0.56	0.85	0.18
*p*-value				
TRT ^3^	0.747	0.001	0.134	0.027
Linear ^4^	0.489	<0.001	0.847	0.039
Quadratic ^4^	0.829	0.815	0.053	0.095

The data are presented as means (*n* = 6). ^1^ Dietary treatments: 0% (C), 0.6% (A-0.6), or 1% (A-1) of macroalgae. ^2^ SEM: standard error of the means. ^3^ TRT: overall effect of dietary treatment. ^4^ Orthogonal polynomial (linear and quadratic) contrasts were used to test the effect of macroalgae level in the diets. ^a,b^ Means within a column with the different superscript are statistically different (*p* < 0.05) based on Tukey’s HSD post hoc test.

**Table 3:** Values of biochemical indicators in piglets’ blood plasma.

Treatment ^1^	TP g/L	ALB g/L	UREA mmol/L	CREAT μmol/L	UA mmol/L	BIL μmol/L
C	91.75 ^a^	58.43 ^a^	5.22 ^a^	121.7	0.432	9.19
A-0.6	65.45 ^c^	44.06 ^b^	4.85 ^a^	122.1	0.441	9.25
A-1	74.71 ^b^	54.13 ^a^	2.78 ^b^	113.5	0.414	11.57
SEM ^2^	2.86	1.67	0.277	1.98	0.010	0.767
*p*-value						
TRT ^3^	<0.001	<0.001	<0.001	0.129	0.520	0.376
Linear ^4^	<0.001	0.002	<0.001	0.169	0.620	0.321
Quadratic ^4^	<0.001	<0.001	0.048	0.955	0.772	0.902

TP: total protein; ALB: albumin; UREA: urea; CREAT: creatinine; UA: uric acid; BIL: bilirubin. The data are presented as means (*n* = 6). ^1^ Dietary treatments: 0% (C), 0.6% (A-0.6), or 1% (A-1) of macroalgae. ^2^ SEM: standard error of the means. ^3^ TRT: overall effect of dietary treatment. ^4^ Orthogonal polynomial (linear and quadratic) contrasts were used to test the effect of macroalgae level in the diets. ^a–c^ Means within a column with the different superscript are statistically different (*p* < 0.05) based on Tukey’s HSD post hoc test.

**Table 4:** Activity of selected enzymes in piglets’ blood plasma.

Treatment ^1^	ALP U/L	ALT U/L	AST U/L	LDH U/L	GTP U/L
C	231.6 ^b^	24.29 ^b^	52.07 ^b^	1020.2 ^b^	42.37 ^a^
A-0.6	189.3 ^ab^	38.14 ^a^	65.79 ^a^	852.3 ^c^	28.46 ^b^
A-1	254.2 ^a^	34.99 ^a^	71.42 ^a^	1301.2 ^a^	29.21 ^b^
SEM ^2^	7.27	1.48	2.25	45.88	1.79
*p*-value					
TRT ^3^	<0.001	<0.001	<0.001	<0.001	<0.001
Linear ^4^	0.596	<0.001	<0.001	<0.001	<0.001
Quadratic ^4^	<0.001	<0.001	<0.001	<0.001	<0.001

ALP: alkaline phosphatase; ALT: alanine aminotransferase; AST: aspartate aminotransferase; LDH: lactate dehydrogenase; GTP: gamma-glutamyltransferase. The data are presented as means (*n* = 6). ^1^ Dietary treatments: 0% (C), 0.6% (A-0.6), or 1% (A-1) of macroalgae. ^2^ SEM: standard error of the means. ^3^ TRT: overall effect of dietary treatment. ^4^ Orthogonal polynomial (linear and quadratic) contrasts were used to test the effect of macroalgae level in the diets. ^a–c^ Means within a column with the different superscript are statistically different (*p* < 0.05) based on Tukey’s HSD post hoc test.

**Table 5:** Lipid indicators in piglets’ blood plasma.

Treatment ^1^	TG mmol/L	TCH mmol/L	HDL mmol/L	LDL mmol/L	%HDL %
C	1.27	3.81 ^a^	1.85 ^b^	1.38 ^a^	48.89 ^b^
A-0.6	1.07	3.34 ^b^	1.87 ^b^	0.977 ^b^	56.12 ^a^
A-1	1.11	3.70 ^a^	2.30 ^a^	0.892 ^b^	62.46 ^a^
SEM ^2^	0.048	0.066	0.060	0.069	1.687
*p*-value					
TRT ^3^	0.237	0.003	<0.001	0.003	0.001
Linear ^4^	0.121	0.059	<0.001	0.001	<0.001
Quadratic ^4^	0.110	0.001	0.543	0.003	0.009

TG: triglycerides; TCH: total cholesterol; HDL: high-density lipoprotein cholesterol; LDL: low-density lipoprotein cholesterol; %HDL: the percentage of HDL in relation to total cholesterol. The data are presented as means (*n* = 6). ^1^ Dietary treatments: 0% (C), 0.6% (A-0.6), or 1% (A-1) of macroalgae. ^2^ SEM: standard error of the means. ^3^ TRT: overall effect of dietary treatment. ^4^ Orthogonal polynomial (linear and quadratic) contrasts were used to test the effect of macroalgae level in the diets. ^a,b^ Means within a column with the different superscript are statistically different (*p* < 0.05) based on Tukey’s HSD post hoc test.

**Table 6:** Oxidative stress markers in piglets’ blood plasma.

Treatment ^1^	LOOH μmol/L	MDA μmol/L	FRAP μmol/L	CAT U/mL	GSH μmol/L	Ascorbic Acid μmol/L	SOD U/mL
C	2.58 ^a^	5.68 ^a^	7.49 ^b^	2.31	0.833 ^c^	27.64	10.84 ^b^
A-0.6	2.28 ^a^	4.94 ^a^	9.48 ^a^	2.28	1.14 ^b^	26.60	11.13 ^b^
A-1	1.75 ^b^	3.56 ^b^	10.55 ^a^	2.38	1.78 ^a^	28.48	12.55 ^a^
SEM ^2^	0.088	0.253	0.354	0.063	0.099	0.357	0.211
*p*-value							
TRT ^3^	<0.001	<0.001	<0.001	0.827	<0.001	0.092	<0.001
Linear ^4^	<0.001	<0.001	<0.001	0.775	<0.001	0.682	<0.001
Quadratic ^4^	<0.001	0.028	<0.001	0.897	<0.001	0.253	0.175

LOOH: lipid hydroperoxide; MDA: malondialdehyde; FRAP: ferric-reducing ability of plasma; CAT: catalase; GSH: reduced glutathione; SOD: superoxide dismutase. The data are presented as means (*n* = 6). ^1^ Dietary treatments: 0% (C), 0.6% (A-0.6), or 1% (A-1) of macroalgae. ^2^ SEM: standard error of the means. ^3^ TRT: overall effect of dietary treatment. ^4^ Orthogonal polynomial (linear and quadratic) contrasts were used to test the effect of macroalgae level in the diets. ^a–c^ Means within a column with the different superscript are statistically different (*p* < 0.05) based on Tukey’s HSD post hoc test.

**Table 7:** Oxidative stress markers in the liver and ileum of piglets.

Treatment ^1^	LOOH μmol/g	MDA μmol/g	CAT U/g	GSH μmol/g	SOD U/g	LOOH μmol/g	MDA μmol/g	CAT U/g	GSH μmol/g	SOD U/g
	Liver					Ileum				
C	8.57 ^a^	11.80 ^a^	95.08	1.04 ^c^	200.9 ^c^	3.86 ^a^	6.48 ^a^	81.99 ^c^	0.863 ^c^	145.6 ^b^
A-0.6	8.31 ^a^	11.08 ^a^	96.02	1.45 ^b^	226.1 ^b^	3.74 ^a^	5.93 ^a^	92.30 ^b^	1.19 ^b^	150.7 ^a^
A-1	6.88 ^b^	8.18 ^b^	94.46	1.73 ^a^	237.9 ^a^	2.98 ^b^	4.28 ^b^	97.10 ^a^	1.45 ^a^	149.0 ^ab^
SEM ^2^	0.261	0.426	0.306	0.073	4.09	0.130	0.261	1.67	0.059	0.754
*p*-value										
TRT ^3^	0.008	<0.001	0.103	<0.001	<0.001	0.003	<0.001	<0.001	<0.001	0.011
Linear ^4^	0.005	<0.001	0.516	<0.001	<0.001	0.003	<0.001	<0.001	<0.001	0.020
Quadratic ^4^	0.193	0.023	0.052	0.258	0.089	0.131	0.084	0.089	0.217	0.018

LOOH: lipid hydroperoxide; MDA: malondialdehyde; CAT: catalase; GSH: reduced glutathione; SOD: superoxide dismutase. The data are presented as means (*n* = 6). ^1^ Dietary treatments: 0% (C), 0.6% (A-0.6), or 1% (A-1) of macroalgae. ^2^ SEM: standard error of the means. ^3^ TRT: overall effect of dietary treatment. ^4^ Orthogonal polynomial (linear and quadratic) contrasts were used to test the effect of macroalgae level in the diets. ^a–c^ Means within a column with the different superscript are statistically different (*p* < 0.05) based on Tukey’s HSD post hoc test.

## Data Availability

The original contributions presented in the study are included in the article, and further inquiries can be directed to the corresponding author(s).
